# Integrating Nanotechnological Advancements of Disease-Modifying Anti-Rheumatic Drugs into Rheumatoid Arthritis Management

**DOI:** 10.3390/ph17020248

**Published:** 2024-02-14

**Authors:** Sukhbir Singh, Neha Tiwary, Neelam Sharma, Tapan Behl, Anita Antil, Md. Khalid Anwer, Seema Ramniwas, Monika Sachdeva, Gehan M. Elossaily, Monica Gulati, Shreesh Ohja

**Affiliations:** 1 Department of Pharmaceutics, MM College of Pharmacy, Maharishi Markandeshwar (Deemed to be University), Mullana-Ambala 133207, Haryana, India; singh.sukhbir12@gmail.com (S.S.); nehatiwary1206@gmail.com (N.T.); neelam.mdu@gmail.com (N.S.); 2 Amity School of Pharmaceutical Sciences, Amity University, Mohali 140306, Punjab, India; 3 Janta College of Pharmacy, Butana, Sonepat 131302, Haryana, India; anitaantilpharma99@gmail.com; 4 Department of Pharmaceutics, College of Pharmacy, Prince Sattam Bin Abdulaziz University, Al-Kharj 11942, Saudi Arabia; m.anwer@psau.edu.sa; 5 University Centre for Research and Development, Department of Biotechnology, Chandigarh University, Gharuan, Mohali 140413, Punjab, India; seema.ramniwas@gmail.com; 6 Fatimah College of Health Sciences, Al-Ain P.O. Box 24162, United Arab Emirates; monikasachdeva@rediffmail.com; 7 Department of Basic Medical Sciences, College of Medicine, AlMaarefa University, P.O. Box 716666, Riyadh 11597, Saudi Arabia; jabdelmenam@um.edu.sa; 8 School of Pharmaceutical Sciences, Lovely Professional University, Phagwara 1444411, Punjab, India; monicagulati14@gmail.com; 9 ARCCIM, Faculty of Health, University of Technology Sydney, Ultimo, NSW 20227, Australia; 10 Department of Pharmacology and Therapeutics, College of Medical and Health Sciences, United Arab Emirates University, Al Ain P.O. Box 15551, United Arab Emirates

**Keywords:** DMARDs, nanotechnology, drug delivery, nanoparticles, nanomicelles, rheumatoid arthritis, solid lipid nanoparticles, treatment

## Abstract

Disease-modifying anti-rheumatic drugs (DMARDs) is a class of anti-rheumatic medicines that are frequently prescribed to patients suffering from rheumatoid arthritis (RA). Methotrexate, sulfasalazine, hydroxychloroquine, and azathioprine are examples of non-biologic DMARDs that are being used for alleviating pain and preventing disease progression. Biologic DMARDs (bDMARDs) like infliximab, rituximab, etanercept, adalimumab, tocilizumab, certolizumab pegol, and abatacept have greater effectiveness with fewer adverse effects in comparison to non-biologic DMARDs. This review article delineates the classification of DMARDs and their characteristic attributes. The poor aqueous solubility or permeability causes the limited oral bioavailability of synthetic DMARDs, while the high molecular weights along with the bulky structures of bDMARDs have posed few obstacles in their drug delivery and need to be addressed through the development of nanoformulations like cubosomes, nanospheres, nanoemulsions, solid lipid nanoparticles, nanomicelles, liposome, niosomes, and nanostructured lipid carrier. The main focus of this review article is to highlight the potential role of nanotechnology in the drug delivery of DMARDs for increasing solubility, dissolution, and bioavailability for the improved management of RA. This article also focusses on the different aspects of nanoparticles like their applications in biologics, biocompatibility, body clearance, scalability, drug loading, and stability issues.

## 1. Introduction

Rheumatoid arthritis (RA) is an autoimmune condition that gradually destroys articular cartilage and can cause abnormalities in vascular, metabolic, bone, and psychological function [1,2,3]. The proximal interphalangeal, wrists, cervical spine, metatarsophalangeal, and metacarpophalangeal joints are recurrently affected joints in RA [4]. Various risk factors causing RA include smoking [5], menopause [6], hormonal disturbance [7,8,9], periodontal disease [10,11,12], and human leukocyte antigen (HLA), namely, HLA-DRB1 shared epitope alleles [13,14].

Nowadays, disease-modifying anti-rheumatic drugs (DMARDs) and biologic DMARDs (bDMARDs) are recommended for the treatments of RA. The poor aqueous solubility or permeability causes the limited oral bioavailability of synthetic DMARDs, while the high molecular weights along with the bulky structures of bDMARDs have posed few obstacles in their drug delivery [15,16,17]. These challenges can be addressed through the development of nanoformulations like cubosomes, nanospheres, nanoemulsions, solid lipid nanoparticles, nanomicelles, liposome, niosomes, and nanostructured lipid carrier.

This review article briefly highlights the physicochemical and pharmacological characteristics of DMARDs. With this viewpoint, nanoformulations that have been developed in the previous decades for the purpose of increasing solubility, in vitro dissolution, and in vivo bioavailability are summarized in this review. This article provides a concise overview of the several facets of nanoparticles, including their utilization in biologics, compatibility with biological systems, elimination from the body, capacity for scalability, drug incorporation, and concerns about stability. The patents and clinical status of DMARDs are also outlined in this review.

## 2. DMARDS: Classification and Characteristics

**Figure 1:**
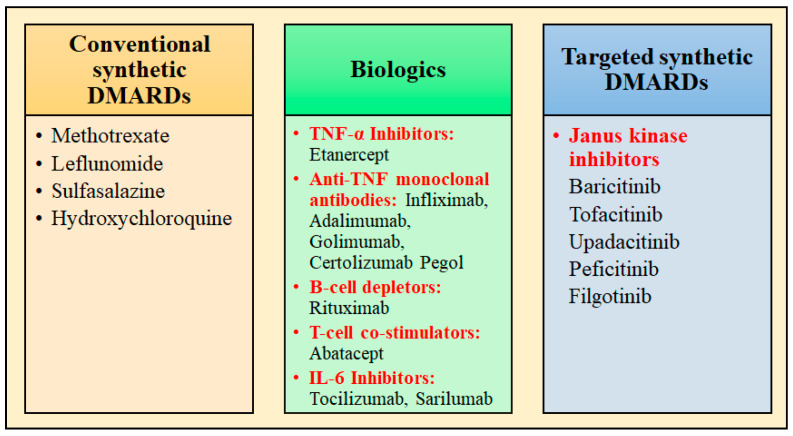
Classification of DMARDs used for the management of rheumatoid arthritis.

DMARDs are the drugs prescribed for treating RA and are divided into three categories, as depicted in Figure 1. In contrast to NSAIDs, these slower-acting DMARDs not only reduce symptoms but also halt the development of clinical manifestations of RA. During the initial phase of disease management with DMARDs, rapid-acting drugs like NSAIDs and glucocorticoids are frequently prescribed as “bridge” therapies because of their facilitation of the delay in disease onset, from few weeks to months [18].

**Figure 2:**
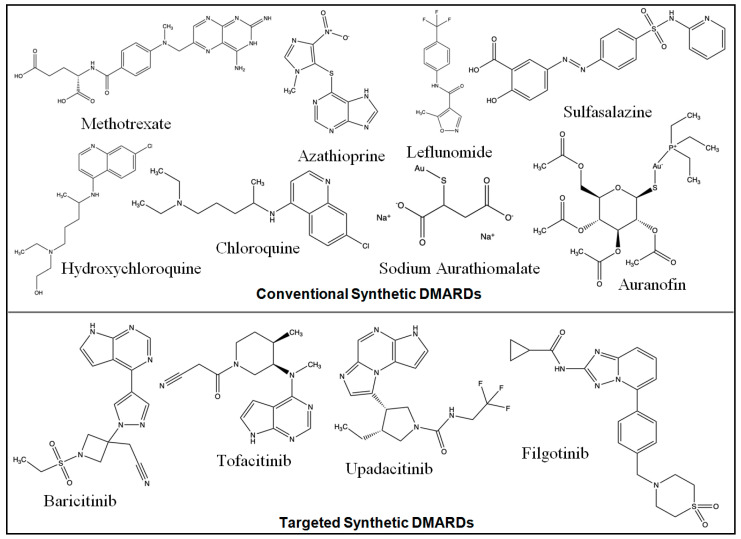
Chemical structures of conventional and targeted synthetic DMARDs.

The examples of conventional synthetic DMARDs are azathioprine, sulfasalazine, leflunomide, intramuscular gold, methotrexate (MTX) (oral and subcutaneous), and antimalarials (hydroxychloroquine/chloroquine). Increased adenosine discharge from fibroblasts, decreased neutrophil adhesion, the decreased production of leukotriene B4 through neutrophils, the reduced levels of local IL-1 as well as IL-6 and IL-8, the inhibition of cell-mediated immunity, and the reduction in synovial collagenase gene expression are all effects of MTX [19]. The chemical structures of synthetic DMARDs are provided in Figure 2, and the description of the characteristics and pharmacological properties of DMARDs is provided in Table 1.

Etanercept is a TNF-receptor antagonist that functions by attaching to TNF-α and TNF-β, which are cytokines implicated in inflammatory response, therefore obstructing their interaction with TNF-receptor [47]. Certolizumab, adalimumab, golimumab, and infliximab are anti-TNF monoclonal antibodies, which effectively neutralizes the biological effects of TNF by tightly binding to both the soluble and transmembrane forms of TNF, therefore preventing TNF from interacting to its receptors [48]. T-cell co-stimulators like abatacept; B-cell depleting drug, e.g., rituximab; and IL-6 inhibitors (tocilizumab, sarilumab) are also examples of biologic DMARDs, which act differently through the inhibition of important pathways in inflammatory cascade [19,49]. Janus kinase inhibitors are the targeted synthetic DMARDs, which include Baricitinib, Tofacitinib, and Upadacitinib. The description of the characteristics and pharmacological properties of biologic DMARDs is provided in Table 2.

## 3. Potential Role of Nanotechnology in Delivery of DMARDs

DMARDs have the potential to slow down the advancement of RA, therefore mitigating the risk of permanent harm to joints and adjacent tissues. Although traditional treatment may provide a degree of therapeutic efficacy, it is accompanied by significant risks such as therapeutic intolerance and dose-dependent side effects. It is essential to provide patients with advanced treatment approaches that effectively mitigate adverse effects [57,58]. The physicochemical characteristics of DMARDs like poor aqueous solubility or poor permeability causes the limited oral bioavailability of synthetic DMARDs, while the high molecular weights along with the bulky structures of bDMARDs have posed few obstacles in their drug delivery and need to be addressed through the development of nanoformulations. Nanoparticles serve as an innovative mechanism for delivering drugs, as they can be designed to effectively target specific cells and tissues. The nanoparticles possess greater drug-loading capacity, which results in an improvement in the pharmacokinetic profile and the ability to deliver drugs safely and effectively. Furthermore, the nanoparticles contribute to an increase in the oral bioavailability of therapeutic agents. Drug-loaded nanoparticles offer various advantages in comparison to the traditional drugs. These benefits include improved insoluble drug delivery, targeted cell recognition, fewer systemic side effects, protection against drug deterioration, and controlled drug release. They also include the improvement of drug diffusion across biomembranes and the incorporation of diagnostic tools as therapeutic agents. Depending on the composition of the matrix, nanoparticles typically range in size from 10 to 1000 nm and have a variety of surface, mechanical, and physicochemical features. Additionally, these particles possess diverse surface features, as well as mechanical and physicochemical characteristics. Extensive research has been conducted on the use of nanoparticles for delivering drugs in the therapeutic management of several medical conditions. Numerous investigations have been dedicated to examining the utilization of nanoparticles within the field of autoimmunity [58,59]. The reason for this phenomenon is in the ability of nanoparticles to be produced with a high degree of selectivity towards certain cells, thus facilitating a controlled and gradual release of DMARDs. This controlled release mechanism not only mitigates the risk of systemic toxicity but also enhances the distribution of these therapeutic agents throughout the body [60]. The application of the current RA treatment is generally limited by the nonselective action of drugs, necessitating dose escalation. Nanotechnology-based techniques have been shown to be especially effective in addressing this issue with regard to RA treatment. The reason for this phenomenon is attributed to the ability of nanoparticulate systems to mitigate the adverse effects of chemotherapeutic drugs, while simultaneously increasing their therapeutic effectiveness. In general, these therapeutic agents exhibit significant toxicity towards both inflamed and normal cells, hence posing a substantial challenge, since their efficacy may be constrained by their toxic nature. However, by the use of various approaches, such as passive and active targeting, the encapsulation of these drug substances into nanoparticles has the potential to enhance their selectivity towards inflammatory cells and tissues [61]. The nanoformulations like cubosomes, nanospheres, nanoemulsions, solid lipid nanoparticles, nanomicelles, liposome, niosomes, and nanostructured lipid carrier that have been developed in the previous decades for the purpose of increasing solubility, in vitro dissolution, and in vivo bioavailability are summarized in Table 3, and the brief description of the important attributes of these nanocarriers are discussed in the subsequent subsections.

### 3.1. Types of Nanoparticles Explored for Drug Delivery of DMARDs

#### 3.1.1. Cubosomes

The cubosomes are self-assembled liquid crystalline particles of a certain emulsifier that have the ideal amount of water in them and an architecture that provides unique functionalities. Cubosomes are biodegradable as well as non-toxic and can solubilize a variety of hydrophilic, hydrophobic, and amphiphilic molecules. These are three-dimensional honeycomb-like structures made of the curved bicontinuous bilayers of lipids that are isolated into two interior aqueous channels. Due to their unique characteristics, including thermodynamic integrity, bioadhesion, and the ability to provide the regulated release by functionalization, cubosomes are perceived to be intriguing vehicles for a variety of administration methods [62].

#### 3.1.2. Nanospheres

Nanospheres are characterized as spherical particles with 10–200 nm diameters in which drug are entrapped or encapsulated into polymeric matrix [63]. In the polymer matrix system, the drug is distributed in a physically and evenly dispersed manner. Nanospheres have the capacity to exist in either an amorphous or a crystalline state, and exhibit the capability of protecting the drug from chemical and enzymatic degradation [64].

#### 3.1.3. Nanoemulsions

Nanoemulsions are innovative drug delivery technology that enables the controlled and sustained release of drugs, biologically active compounds, and genetic material. A nanoemulsion is a stable liquid solution composed of oil, surfactant, and aqueous phase. It exhibits isotropic clarity and thermodynamic or kinetic stability, often characterized by droplet sizes ranging from 10 to 500 nm [65,66,67]. The use of nanoemulsion facilitates enhanced drug absorption and targeting due to the presence of nanoscale droplets. This advancement not only enhances the traditional emulsion systems but also presents novel prospects for the development of pharmaceuticals with improved precision in terms of bioavailability and dosage accuracy, hence minimizing adverse effects. Nanoemulsions are widely used in many biomedical applications because of their tiny droplet sizes, which provide outstanding features such as strong stability and adjustable rheology. Nanoemulsions are commonly used in development of pharmaceutical formulations for topical, ocular, intravenous, and other modes of delivery [68,69,70].

#### 3.1.4. Solid Lipid Nanoparticles (SLNs)

SLNs are currently a cutting-edge technology used in the developing field of nanotechnology as a result of their numerous potential applications in medication delivery, clinical care, research, and other science disciplines. SLNs comprise of lipids like triglycerides that are solid at normal temperature. These lipids include hydrophobic core that enables the dissolution or dispersion of drugs inside them [71,72]. Lipid nanoparticles possess distinct size-dependent characteristics, hence presenting an exciting potential for novel therapeutic approaches. The smaller dimensions and the lipophilic characteristics of these nanoparticles facilitate their diffusion through cell membranes [73]. SLNs are also used for the purposes of nanotheranostics. SLNs possess a lipid core, which enables them to exhibit enhanced loading capacity for weakly water-soluble medicines and imaging probes. The encapsulation of drugs into nanocarriers is an innovative approach in the field of drug delivery, which holds promise for the application of the advanced strategies of drug targeting. SLNs have significant prospects in the targeted delivery of drugs, thereby gaining considerable interest among researchers [74].

#### 3.1.5. Nanomicelles

Nanomicelles are nanoscale self-assembling colloidal dispersions with core–shell structure, having dimension in range of 10–100 nm. These nanostructures are fabricated using amphiphilic blocks in which the core is composed of hydrophobic blocks like propylene oxide and PLGA, while the outer shell comprises hydrophilic blocks like polyethylene glycol and polyvinyl alcohol. Nanomicelles have the capability to entrap hydrophobic drugs and imaging agents inside their core, hence avoiding the need for the utilization of hazardous organic solvents [75]. Because of few unique factors like their dimensions, solubility characteristics, customized surface properties, and interaction with surrounding environment, nanomicelles have emerged as distinctive and innovative nanomaterials [76]. The properties of nanomicelles, as a versatile tool, are beneficial in biological applications. The size and shape of micelles are reliant upon the molecular structure of surfactant and the prevailing characteristics of the solution, including pH, temperature, ionic strength, and surfactant concentration [77].

#### 3.1.6. Liposome

Liposomes have been comprehensively investigated as nanocarriers in the targeted drug delivery of hydrophobic and hydrophilic drugs with potential therapeutic activities. They have unique properties like excellent biocompatibility, biodegradability, and minimal immunogenicity [78]. Liposomes possess an aqueous core that is encompassed by a bilayer composed of phospholipids. Liposomes have shown the ability to improve drug solubility and regulate distribution, in addition to their potential for surface changes to provide targeted, extended, and sustained release. Liposomes are synthetic vesicles that possess a spherical shape, often exhibiting size in range of 50–500 nm in diameter. Cholesterol and non-toxic phospholipids from natural sources make up the architecture of liposomes. The size, the capacity to encapsulate both hydrophobic and hydrophilic medicines, and the biocompatibility of liposomes make them promising drug delivery platforms [79,80].

#### 3.1.7. Niosomes

Niosomes are lipid-based vesicles made of excipients like cholesterol and non-ionic surfactants. In the realm of medication delivery, these structures are applied to target particular regions and achieve intended therapeutic outcomes. Niosomes typically have a size distribution between 10 and 1000 nanometers. The basic composition of niosomes closely resembles that of liposomes, since both have bilayer membrane structure that encloses aqueous compartment. In contrast to the bilayer’s phospholipid components, niosomes are created by utilizing a variety of nonionic surfactants, such as spans and tweens [81,82]. Similar to the liposome formulation, their structure encourages the creation of a membrane bilayer after an exposure to an aqueous molecule. Niosomes are made with cholesterol in order to increase the bilayer membrane’s stiffness. When creating niosomes, other components, such as charging lipids, may be employed to provide the nanoparticles a specific surface charge. The use of nonionic surfactants in the production of niosomes has many benefits over liposomes, particularly in terms of cost-effectiveness and stability [83]. These are flexible nanoparticles, owing to their ability to contain hydrophilic medicines within their aqueous core and lipophilic medications in their outer bilayer [84,85,86].

#### 3.1.8. Nanostructured Lipid Carrier (NLCs)

NLCs are second-generation lipid carriers that have the potential to address the limitations associated with solid lipid nanoparticles. These NLCs have found use in a diverse range of treatment strategies [87]. NLCs have a less organized lipidic core because they combine solid and liquid lipids. This intrinsic design flaw makes it easier to accommodate more drugs. The NLCs outweigh SLNs because they can encapsulate larger amounts of medicine, contain less water, and provide better drug entrapment with less leakage during storage [88,89]. The emergence of lipids as a promising drug delivery method is due to their biocompatibility [90]. It was shown that NLCs improved conventional carriers in a number of ways, like increased permeability, higher bioavailability, fewer side effects, extended half-life, and tissue-targeted delivery [91].

**Table 3:** The summary of research outcomes about in vitro/in vivo studies of nanoparticles formulations of DMARDs.

Formulation	Method of Preparation	Excipients	Outcomes	Ref.
Methotrexate (MTX)				
Teriflunomide and MTX-loaded hydroxyapatite nanoparticles (HAP-NP)	Wet chemical precipitation method	Calcium nitrate tetrahydrate, ammonium dihydrogen phosphate, and cetyltrimethylammonium bromide	The in vitro release of TEF and MTX from NP was 70.41 ± 1.22% and 82.43 ± 1.31% till 24 h, which showed sustained release behavior. An in vivo study showed that NPs exhibited significant decrease in ankle diameter and arthritis score and showed the least hepatotoxicity. Biochemical investigations showed insignificant changes in glutamic oxaloacetate transaminase and glutamic pyruvic transaminase levels.	[92]
Self-assembled nanoparticles	Counter-ion induced gellification	Glycol chitosan, steric acid, sodium alginate, and calcium chloride	In vitro MTX release from NPs illustrated sustained drug release till 24 h. NPs showed intercellular uptake in murine macrophage cells, i.e., RAW 264.7, using confocal microscopy and FACS analysis. In vivo study in collagen-induced arthritis mice showed the significant accumulation of NPs in inflamed joints and demonstrated significantly higher therapeutic activity in comparison to free MTX.	[93]
Sodium alginate chitosan nanoparticles	Ionotropic pre-gelation method	Chitosan and sodium alginate	In vitro drug release from NPs showed the initial burst release and then sustained release of 68.99% till 36 h.	[94]
Multifunctional folate receptor-targeting and pH-responsive nanocarriers (MTX-loaded FA-PPLNPs)	Modified emulsion–solvent evaporation	PLGA, lipopolysaccharide, folic acid, polyethylene glycolpoly (lactic-co-glycolic acid), and poly (cyclohexane-1,4-diylacetone dimethylene ketal)	In vitro drug release from NPs showed the burst release of 14% and 35% MTX within 1 h, 35% and 62% till 6 h, and 64% and 81% till 36 h, at pH 7.4 and 5, respectively. Cellular uptake in RAW246.7 cells and cytotoxicity study using the MTT assay of NPs revealed superior cellular uptake and higher cytotoxicity, which might be attributable to their active targeting on activated macrophages. In vivo study in adjuvant-induced arthritis rat model revealed that the average clinical score (0.6) and paw thickness (6.18 mm) of NPs-treated rats were nearly similar as those of normal rats.	[95]
Cubosomes	Lipid emulsification coupled with high-pressure homogenization technique	Poloxamer 188	The in vitro study of cubosomes revealed sustained drug release for 12 h. Ex vivo skin permeation using the excised skin of Wistar rats demonstrated 2.50 ± 0.3 ng of MTX permeation within 2 h and 8.80 ± 5.2 ng within 12 h. In vivo study using rat tail flick method showed that thermal stimulus time was 5.63 ± 0.21 s and 2.70 ± 0.20 s with drug-loaded cubosomes and diclofenac gel, which showed the higher analgesic activity of cubosomes. The paw thickness in complete Freund’s adjuvant (CFA)-induced arthritic rats was reduced from 1.47 cm to 1.03 cm within 15 days in cubosome-treated rats.	[96]
MTX and gold nanoparticle-loaded multifunctional temperature-responsive nanospheres	Emulsion–diffusion evaporation technique	PEG-PLGA and poly (vinyl alcohol)	The in vitro drug release profile of NPs in PBS (pH 7.4) was a sustained release pattern till 120 h. In vitro cytotoxicity assessed using the MTT assay in THP1 monocytes and differentiated macrophages showed significant improvement in the cytotoxic effect in the presence of Au-NPs in nanospheres. In vitro anti-inflammatory activity assessed using cytokines measurement showed that nanospheres significantly decreased the levels of IL-1, IL-6, and TNF-α in THP-1 monocytes and differentiated macrophages.	[97]
MTX and superparamagnetic iron oxide nanoparticles (SPION) Co-associated into PLGA nanoparticles conjugated with anti-CD64 antibody	Solvent emulsification–evaporation method	PLGA	In vitro MTT and LDH assays were performed with RAW 264.7 cells to study the effect of NPs on cell viability and cytotoxicity. It was found that, after 24 h of incubation, the toxicity of MTX-loaded NPs was higher than the free drug.	[98]
Lipid nanoemulsions	High pressure homogenization	Cholesteryl oleate, egg phosphatidylcholine, cholesterol, and tween 80	In vivo study in antigen-induced arthritis (AIA) rabbits revealed that animals treated with the intraarticular injection of lipid nano-emulsion showed reductions in synovial leukocyte infiltrate and protein leakage in comparison to those of non-treated arthritic rats.	[99]
MTX-loaded PLGA Au half-shell nanoparticles conjugated with arginine–glycine aspartic acid (RGD) peptides over the surface of gold half-shell	Nanoprecipitation method	PLGA, carboxylic acid, Au, and EDC	The in vivo study of developed NPs was executed in collagen-induced arthritic mice, which showed that NPs when injected into arthritic mice effectively delivered the drug to inflamed joints due to the presence of RGD peptides over NPs. Upon near-infrared irradiation exposure, heat was produced by gold half-shells, which leads to rapid drug release from PLGA nanoparticles.	[100]
Liposomal MTX (MTX-gamma-DMPE)	Hydration method	Egg lecithin, cholesterol and phosphatidic acid, distearoylphosphatidylcholine, polyethylene glycol, and DMPE	The in vitro study of liposomes was performed by the estimation of cytokine production by macrophages, which showed that liposomes caused the inhibition of IL-1 and PGE2. An in vivo study was performed in collagen-induced arthritis in Lewis rats, and treatments to different groups of rats were provided intravenously. The clinical score and hind paw diameter measurements remained significantly lower in MTX-loaded liposomes along with the reduced side effects.	[101]
Sulfasalazine (SSZ)				
Nanoparticles	Nanoprecipitation and ionotropic gelation techniques	Eudragit S100 and ethyl cellulose	Nanoprecipitation was found to be a comparatively better technique for the preparation of SSZ-NPs, which produced a mean particle diameter of 165.4 nm, the zeta potential of −47.7 mV, the entrapment efficiency of 89.29%, and could sustain drug release for 12 h in the in vitro study.	[102]
Micellar/liposomal Micellar/niosomal	Solvent evaporation method, thin film hydration method, followed by sonication	Soy lecithin, tween 80, squalene, and polyvinyl alcohol	The in vitro release study of liposomes showed that slow drug release was 25% at 10 days and 50% at 30 days, while niosomes exhibited ~40% release at 10 days. The toxicity of SSZ nano-formulations against human dermal fibroblasts was assessed using the MTT viability assay. The IC50 of SSZ was decreased by about 4-folds from 940 mM for free SSZ to 240 mM for liposomal or 230 mM for niosomal SSZ.	[103]
Leflunomide				
Nanostructured lipid carriers (NLC)	Melt emulsification ultrasonication method	Stearic acid, oleic acid, tween 80, and poloxamer 188	The in vitro drug release study of NLCs (F1) in phosphate-buffered saline of pH 7.4 exhibited 90.35% drug release in 48 h. In vivo anti-inflammatory activity was examined in CFA-induced arthritic Sprague Dawley rats, which revealed that NLCs exhibited great potential in decreasing CFA-induced knee edema over thirty days of treatment. In vivo intestinal lymphatic uptake study in Sprague Dawley rats showed that NLCs produced an increase in lymphatic drug uptake, which might be due to chylomicron formation.	[104]
Superparamagnetic iron oxide nanoparticles (SPION) bioemulsomes	Thin-film hydration method	L-α-phosphatidylcholine (Lipoid^®^ S100), cholesterol, compritol 888 ATO^®^ CA, ferric chloride hexahydrate, ferrous sulfate tetrahydrate, and ammonium hydroxide	An in vitro study showed that bioemulsomes exhibited a two-phase release pattern with the initial burst release in the first 1 h and sustained release for 24 h. The in vivo study revealed that the intra-articular injection of bioemulsomes for 14 days in CFA-induced arthritic Sprague Dawley rats showed a normal joint diameter after 14 days of treatment, with statistically insignificant difference compared to the negative control.	[105]

CFA: complete Freund’s adjuvant; DMPE: dimyristoyl phosphatidylethanolamine; EDC: 1-Ethyl-3-(3-dimethylaminopropyl) carbodiimide; MTX: methotrexate; PEG: polyethylene glycol; PLGA: poly (lactic-co-glycolic acid); and SPION: superparamagnetic iron oxide nanoparticles.

### 3.2. Promising Developments of Nanoparticles-Based Drug Delivery Systems

The use of nanoparticles has been extensive for enhancing the pharmacokinetic and pharmacodynamic properties of medications, primarily by augmenting the bioavailability of pharmaceuticals with limited solubility. Conversely, this might also heighten the likelihood of adverse effects arising, perhaps leading to unsafe levels, which might cause the failure of nanoparticles in drug delivery. Nevertheless, the nanoparticles may undergo surface modification to enhance their specificity and therapeutic effectiveness by conjugating with antibodies, peptides, or polysaccharides that specifically target the receptors present in the afflicted tissue of RA. By integrating passive and active targeting techniques, medication delivery may be optimized, resulting in reduced toxicity and undesired side effects, ultimately enhancing patient outcomes, and, thus, imparting success to the nanoparticles’ drug delivery approach [61,106].

### 3.3. Why Do Nanoparticles Outperform Conventional Delivery Methods?

The traditional medicines for RA have several limitations, including low patient compliance, the short duration of action, limited absorption into the body, and poor solubility. These issues can potentially be addressed by exploring nanoparticle-based therapies, which have the ability to enhance drug effectiveness by delivering the medication in higher concentrations to the desired site. As a result, nanoparticles surpass the conventional delivery methods [107,108]. The distinguishing characteristic of nanosized material, in contrast to bulk material, is the benefit of a higher surface-to-volume ratio [109,110]. Nanotechnology has the potential to address the drawbacks of traditional delivery methods, ranging from broad concerns like biodistribution to more specific obstacles like intracellular trafficking. This may be achieved by targeted administration to specific cells, molecular transport to certain organelles, and other innovative strategies. Nanoparticles has the capacity to enhance the stability and solubility of enclosed substances, facilitate their passage across membranes, and extend their circulation duration, hence augmenting safety and effectiveness [111].

### 3.4. Applications of Nanoparticles in Biologics

Several research studies have illustrated that the efficacy of biological drug therapy for RA can be improved by using nano-based drug delivery approaches. The various types of nanocarriers have been utilized in the delivery of biologics for the treatment of RA, i.e., the polymeric nanoparticles of adalimumab, rituximab, and trastuzumab [112], the lipid nanoparticles of TNF-α siRNA [113], polyamidoamine dendrimers functionalized with anti-TNF-α antibody as well as chondroitin sulphate [114], and nanoparticle-loaded hydrogels to target TNF-α [115].

### 3.5. Biocompatibility of Nanoparticles

Assessing the biocompatibility of nanoparticles is essential in drug delivery to assure the safe release of drugs without causing harmful consequences such as cytotoxicity, immunogenicity, thrombogenicity, and carcinogenicity [116]. Biocompatible polymers such as polyethylene glycol, polyvinylpyrrolidone, polyacrylamide, polyvinyl alcohol, and polysaccharides are frequently employed to coat nanoparticles. This coating imparts stealth properties, reducing or preventing undesired interactions with opsonin proteins and uptake by the reticuloendothelial system. Consequently, the nanoparticles have a longer half-life in the bloodstream, facilitating their accumulation at the desired site [117].

### 3.6. Clearance of Nanoparticles from the Body

The majority of uncoated nanoparticles may be promptly eliminated from the bloodstream, since they are readily identified by the reticuloendothelial system, resulting in a significant reduction in targeting effectiveness [118]. The fate of nanoparticles in terms of clearance is determined by their size. For instance, nanoparticles smaller than 600 nm may be used because of their enhanced permeation and retention effect. Nevertheless, particles of a size lower than 6 nm may be efficiently excreted by the kidneys. Within the range of nanoparticles measuring 10–100 nm, their half-life demonstrates a positive correlation with their size. Thus, while contemplating the interactions between nanoparticles and biological organs in terms of size, it is important to consider particle sizes that are suitable for clearance and therapy [119]. The elimination of nanoparticles from the body requires a precise synchronization in time. If nanoparticles are eliminated from the body too rapidly, they will not gather at the tumor location and will be expelled along with their contents in the urine. Conversely, nanoparticles that remain in the body may lead to toxicity associated with medications or nanoparticles in organs like the liver or kidneys, which are responsible for eliminating pharmaceuticals and nanomaterials from the bloodstream [120].

### 3.7. Scalability of Nanoparticles

The process of scaling up of a nanomedicine from the laboratory to the commercial scale involves several components. The involved factors include the inherent properties of the material, the toxicological characteristics related to the size and shape of nanoparticles, and the biodegradability of nanocarriers [121]. The selection of the nanoparticle fabrication method is critical for maximizing time efficiency in the context of pilot size manufacturing [122].

### 3.8. Drug Loading of Nanoparticles and Stability Issues

Three primary methodologies have been devised for creating nanoparticles with a high drug-loading capacity. These include post-loading, where drugs are loaded into nanoparticles that have already been synthesized; co-loading, which involves attaching a drug to a polymer or macromolecule and then assembling drug conjugates; and pre-loading, which entails forming drug nanoparticles initially and subsequently coating them with additional materials [123]. The low drug-loading percentage of most nanomedicines is a significant challenge for their clinical translation. This is mostly a result of obstacles such as expensive manufacturing costs, difficulties in scaling up production while maintaining repeatable qualities, and potential hazardous side effects associated with the nanomaterials [124]. High drug-loading nanoparticles need a minimal quantity to reach the desired therapeutic level. This not only minimizes the possible negative effects of excessive materials but also reduces the production cost of the nanomedicine [125]. Consequently, nanoparticles having a high drug-loading capacity would be optimal for achieving a large drug dosage while minimizing the quantity of carrier material required [123]. The nanoparticles are prone to physical instability due to processes such as sedimentation, agglomeration, crystal development, and chemical reactions. These processes result in the formation of aggregates in a dry state, ultimately causing the nanoparticles to lose their unique nanoscale features [126,127].

## 4. Published Patents and Current Clinical Status of DMARDs

The patent research, from the year 2021 to 2023, in the discipline of RA therapy, with the primary objective of either treating the condition or diminishing the disease progression, was conducted using the World Intellectual Property Organization’s official website (Table 4).

## 5. Conclusions

RA is a chronic condition that currently lacks effective treatment. Research findings have shown that the initial administration of DMARDs has demonstrated efficacy in ameliorating symptoms such as pain, joint degeneration, and functional impairment. The role of nanotechnology-based drug delivery in inflamed locations would be further supported by nanocarriers with a particular binding affinity to these inflammation-related cells. Nanocarriers are superior compared to traditional medication forms for treating RA because they have less systemic side effects and higher therapeutic efficacy. The nanoformulations like cubosomes, nanospheres, nanoemulsions, solid lipid nanoparticles, nanomicelles, liposome, niosomes, and nanostructured lipid carrier have been synthesized, and research outcomes elucidate that modified and targeted nanoformulations can be further explored for increasing the dissolution profile and the bioavailability as well as for achieving the targeted drug delivery of DMARDs.

## 6. Future Perspectives

The effectiveness of the existing therapeutic agents for RA has been found to be limited in achieving remission in some individuals. Additionally, these medications have been linked with a range of adverse effects like systemic organ toxicity, affecting the gastrointestinal tract, skin, and kidneys, and immuno-toxicity, leading to increased susceptibility to infections. In order to address these constraints, nanotechnology has been implemented in the therapy of RA due to its potential to enhance medication stability, facilitate targeted drug administration and release, and ultimately increase therapeutic effectiveness. However, it is important to take into account various factors in order to enhance the rate at which nanomedicine is translated from laboratory to clinical practice for the purpose of treating RA. The fundamental characteristic attributed to nanoparticles is their non-immunogenicity. Because RA is characterized by inflammatory conditions, the induction of further immunogenicity might pose a significant risk to sufferers. Biological nanoparticles like exosomes and lipoproteins have shown great potential as nanocarriers with low immunogenicity due to their ability to be obtained from autologous cells and biofluids. Furthermore, it is essential to thoroughly evaluate the distribution route of a nanomedicine in order to optimize its therapeutic effectiveness. The intra-articular delivery route enables the competent localization of a nanomedicine into a targeted inflamed joint. In order to maximize its retention inside the inflamed joint and increase its therapeutic efficiency following intra-articular delivery, the size and surface features of the nanomedicine can be appropriately altered. However, the regular injection of a nanomedicine into an inflamed joint could potentially increase the susceptibility to infection. Contrarily, the intravenous injection of nanomedicine enables the targeted distribution of therapeutic medicines to inflamed joints throughout the entire body. However, the clearance of intravenously delivered nanomedicine by organs in the mononuclear phagocytic system leads to a reduction in drug concentration at the targeted illness site. Hence, it is essential to conduct more research on novel approaches aimed at bypassing the MPS and prolonging the circulation of nanomedicine. Delivering therapeutic medicines to swollen joints would be more effective as a result. Furthermore, it is crucial to clarify the underlying mechanisms of the aberrant lymphatic networks and uncover novel targets within inflammatory joints. As a result, after being administered intravenously, nanomedicine may more effectively target specific targets at inflammatory joints.

## Figures and Tables

**Table 1:** Description of the characteristics and pharmacological properties of synthetic DMARDs.

Drug	Chemical Formula	Mol. Weight (Daltons)	C_max_; T_max_	Bioavailability	Clearance; Half-Life	BCS Class	Ref.
MTX	C_20_H_22_N_8_O_5_	454.43	479 ± 107 ng/mL; 1–2 h	64–90%	Half-life: 3–10 h	Class IV	[20,21,22,23]
Leflunomide	C_12_H_9_F_3_N_2_O_2_	270.20	T_max_: 6–12 h	83–86%	31 mL/h; 2 weeks	Class II	[20,24,25,26,27]
Sulfasalazine	C_18_H_14_N_4_O_5_S	398.39	6 µg/mL; 6 h	10–30%	1 L/h; 7.6 ± 3.4 h	Class IV	[20,28,29,30,31]
Hydroxychloroquine	C_18_H_26_C_l_N_3_O	335.87	129.6 ng/mL; 3.3 h	67–74%	96 mL/min; 123.5 days	Class I	[20,32,33,34]
Baricitinib	C_16_H_17_N_7_O_2_S	371.42	53.4 ng/mL; 1.5 h	80%	8.9 L/h; 12 h	Class III	[20,35,36,37]
Peficitinib	C_18_H_22_N_4_O_2_	326.4	91–741 ng/mL; 1.1–2.1 h	45.9%	11–14 L/h and 8–10 L/h; 9.9–16.2 h	Class IV	[20,38,39]
Filgotinib	C_21_H_23_N_5_O_3_S	425.51Da	2.15 ug/mL; 0.5 to 5.0 h	80%;	4.45 L/h; 5–6 h	Class II	[20,40]
Tofacitinib	C_16_H_20_N_6_O	312.36	3.6 ng/mL; 0.5–1 h	74%	25.0 L/h; 3 h	Class III	[20,41,42,43,44]
Upadacitinib	C_17_H_19_F_3_N_6_O	380.37	159 ± 45.7 ng/mL; 2–4 h	80%	53.7 L/h; 8–14 h	Class I	[20,45,46]

Mol. Weight: molecular weight.

**Table 2:** Outline of chemical formula, average molecular weight, and pharmacological descriptions of biologic DMARDs used in rheumatoid arthritis.

Biologics	Protein Chemical Formula	Average Molecular Weight	C_max_; T_max_	Bioavailability	Clearance; Half-Life	Ref.
TNF-Receptor Antagonist						
Etanercept	C_2224_H_3475_N_621_O_698_S_36_	51,234.9 Da (monomer)	1.1 µg/L; 69 h	56.9%	160 mL/h; 102 h	[20]
Anti-TNF Monoclonal Antibodies						
Infliximab	C_6428_H_9912_N_1694_O_1987_S_46_	144,190.3 Da	75 µg/mL	79.1%	18.4 mL/h; 7.7–9.5 days	[50]
Adalimumab	C_6428_H_9912_N_1694_O_1987_S_46_	144,190.3 Da	4.7 ± 1.6 μg/M; 131 ± 56 h	64%	12 mL/h; 10–20 days	[20]
Golimumab	C_6530_H_10068_N_1752_O_2026_S_44_	146,943.1937 Da	3.2 ± 1.4 µg/mL; 2–6 days	53%	4.9–6.7 mL/day/kg; 2 weeks	[20,51]
Certolizumab pegol	C_2115_H_3252_N_556_O_673_S_16_	91,000.0 Da	-	80%	9–14 mL/h; 14–21 mL/h; 14 days	[20]
B-Cell Depletors						
Rituximab	C_6416_H_9874_N_1688_O_1987_S_44_	143,859.7 Da	157 ± 46 and 183 ± 55 mcg/mL; 3 days	100% (IV)	0.335 L/day; 22 days	[20]
T-Cell Co-stimulators						
Abatacept	C_3498_H_5458_N_922_O_1090_S_32_	92,300.0 Da (with glycosylation)	292 mcg/mL; 4 days	78.6%	0.23 mL/h/kg; 16.7 days	[52]
IL-6 Inhibitors						
Tocilizumab	C_6428_H_9976_N_1720_O_2018_S_42_	148,000.0 Da	51.3 ± 23.2 µg/mL; 2–3 days	79.5%	12.5 mL/h; 21.5 days	[53,54]
Sarilumab	C_6388_H_9918_N_1718_O_1998_S_44_	150,000.0 Da (143,900 Da in the absence of N-glycosylation in heavy chains)	20.0 ± 9.20 mg/L; 2–4 days	80%	4.3 L/day; 10 days	[55,56]

**Table 4:** The delineation of the patent literature related to the therapeutics of rheumatoid arthritis.

Patent Number	Applicant	Publication Date	Patent Title
CN116327700	Suzhou University	27 June 2023	Methotrexate nano drug-loading system, preparation method thereof and application of methotrexate nano drug-loading system in treatment of rheumatoid arthritis [128]
CN116251106	Zhengzhou University	13 June 2023	Application of combination of mangiferin and methotrexate in preparation of medicine for treating rheumatoid arthritis and protecting liver [129]
EP4119140	Synact Pharma APS	18 January 2023	Combination treatment of arthritic disease [130]
WO2022260546	Uniwersytet Medyczny Im. Piastów Śląskich We Wrocławiu	15 December 2022	A glucose-methotrexate conjugate for use in preventing or treating autoimmune diseases [131]
US20220160712	Chan Zuckerberg Biohub, Inc.The Regents of The University of California	26 May 2022	Methods of treating rheumatoid arthritis and for predicting the response to methotrexate [132]
KR 20210119175 A	The Catholic University of Korea Industry-Academic Cooperation Foundation	5 October 2021	Therapeutic uses of methotrexate-nanoparticle [133]
US20210283250	Chugai Seiyaku Kabushiki Kaisha	16 September 2021	Method for treating rheumatoid arthritis with a human IL-6 receptor antibody and methotrexate [134]
KR1020210108103	Korea Advanced Institute of Science and Technology	2 September 2021	Complex for treating rheumatoid arthritis and manufacturing method thereof [135]
KR1020210059657	Industry-Academic Cooperation Foundation, Yonsei University	25 May 2021	Contrast medium agent for optical imaging for early diagnosis of rheumatoid arthritis [136]
CN112675177	Zhujiang Hospital of Southern Medical University	20 April 2021	Pharmaceutical composition containing inhibitor and methotrexate and preparation method and application of pharmaceutical composition [137]

## Data Availability

Not applicable.

## List of Abbreviations

5-ASA	5-aminosalicylic acid
bDMARD	Biologic DMARD
CFA	Complete Freund’s adjuvant
CQ	Chloroquine
DMARDs	Disease-modifying anti-rheumatic drugs
FLS	Fibroblast-like synoviocytes
HCQ	Hydroxychloroquine
MMNPs	Methotrexate and minocycline-loaded nanoparticles
MTCs	Methotrexate-loaded cubosomes
MTX	Methotrexate
NLC	Nanostructured lipid carriers
RA	Rheumatoid arthritis
RANKL	Receptor activator of nuclear factor kappa beta ligands
SLN	Solid lipid nanoparticle
SPION	Superparamagnetic iron oxide nanoparticles
SSZ	Sulfasalazine
Th17	T-helper 17
TNF	Tumor necrosis factor
